# SARS-CoV-2 RNA-binding protein suppresses extracellular miRNA release

**DOI:** 10.1080/15476286.2025.2527494

**Published:** 2025-07-01

**Authors:** Hyejin Mun, Chang Hoon Shin, Qingxuan Fei, Andrea Estefania Lopez Giraldo, Kyoung-Min Choi, Ji Won Lee, Kyungmin Kim, Kyung-Won Min, Leilei Shi, Mark T. Bedford, Dong-Chan Kim, Yoo Lim Chun, Seonghyun Ryu, Dongin Kim, Jeong Ho Chang, Ryan T. Westrope, Michelle Shay, Edward Nguyen, Junho K. Hur, Abigail Agyenda, Nam Chul Kim, Sung-Ung Kang, Woonghee Lee, Je-Hyun Yoon

**Affiliations:** a Department of Oncology Science, University of Oklahoma, Oklahoma City, OK, USA; b Department of Chemistry, University of Colorado, Denver, CO, USA; c Department of Biology, College of Natural Sciences, Gangneung-Wonju National University, Gangneung-si, Republic of Korea; d Department of Epigenetics and Molecular Carcinogenesis, The University of Texas MD Anderson Cancer Center, Houston, TX, USA; e R&D Center, NOSQUEST Inc, Seongnam, Republic of Korea; f Department of Biochemistry and Molecular Biology, Medical University of South Carolina, Charleston, SC, USA; g Department of Pharmaceutical Sciences, College of Pharmacy, University of Oklahoma Health Sciences Center, Oklahoma City, OK, USA; h Department of Biology Education, Kyungpook National University, Daegu, Republic of Korea; i Hanyang Institute of Bioscience and Biotechnology, Hanyang University, Seoul, Republic of Korea; j Department of Pharmacy Practice and Pharmaceutical Science, College of Pharmacy, University of Minnesota, Duluth, MN, USA; k Neuroregeneration and Stem Cell Programs, Institute for Cell Engineering, Johns Hopkins University School of Medicine, Baltimore, USA; l Department of Neurology, Johns Hopkins University School of Medicine, Baltimore, MD, USA; m Department of Pathology, University of Oklahoma, Oklahoma City, OK, USA

**Keywords:** SARS-CoV-2, Nsp9, miRNA, let-7b, POLR2D

## Abstract

SARS-CoV-2 is the betacoronavirus causing the COVID-19 pandemic. Although the SARS-CoV-2 genome and transcriptome were reported previously, the function of individual viral proteins is largely unknown. Utilizing biochemical and molecular biology methods, we identified that four SARS-CoV-2 RNA-binding proteins (RBPs) regulate the host RNA metabolism by direct interaction with mature miRNA let-7b revealed by Nuclear Magnetic Resonance spectroscopy (NMR). SARS-CoV-2 RBP Nsp9 primarily binds mature miRNA let-7b, a direct ligand of the Toll-like Receptor 7 (TLR7), one of the potential SARS-CoV-2 therapeutics. Nsp9 suppresses host gene expression possibly by promoting let-7b-mediated silencing of a cellular RNA polymerase, POLR2D. In addition, Nsp9 inhibits extracellular release of let-7b and subsequent antiviral activity via TLR7. These results demonstrate that SARS-CoV-2 hijacks the host RNA metabolism to suppress antiviral responses and to shut down cellular transcription. Our findings of how a natural ligand of TLR7, miRNA let-7b, is suppressed by SARS-CoV-2 RBPs will advance our understanding of COVID-19 and SARS-CoV-2 therapeutics.

## Introduction

Severe acute respiratory syndrome coronavirus 2 (SARS-CoV-2) causes Coronavirus disease 19 (COVID-19) [1]. It contains a positive-sense, single-stranded RNA genome of ~30 kb similar to other coronaviruses (CoVs) such as SARS-CoV and Middle East respiratory syndrome coronavirus (MERS-CoV) [2,3]. CoVs have the remarkable ability to infect birds and mammals and to transmit the infection to humans [4]. The CoVs genomic RNA contains two open reading frames (ORFs) producing 11 nonstructural proteins (Nsps) from ORF1b and 15 Nsps from ORF1b by ribosome frame-shift and proteolytic cleavages mediated by viral proteases Nsp3 and Nsp5. Nsp12 contains RNA-dependent RNA polymerase (RdRP) activity, forming 5’ capping of viral RNA with help of Nsp9, escaping conventional surveillance (decapping and RNA degradation) and [5–7] producing negative-sense RNA, which serves as the templates for synthesizing positive-sense genomic RNA (gRNA) and sub-genomic RNAs (sgRNAs). The structural proteins encoded in sgRNAs package gRNA into progeny virions with the help of six accessory proteins. The molecular mechanisms of replication and transcription have been studied in other CoVs but are largely unknown in SARS-CoV-2. Recent transcriptome and epitranscriptome analyses by long-read sequencing mapped the sgRNAs, ORFs, and transcription-regulatory sequences (TRSs) along with numerous unconventional RNA joining and modification [8].

Current COVID-19 therapeutics are limited due to frequent mutations in RNA genomes during rapid transmissions [9–11]. Although Lopinavir, Ribavirin and Favipiravir have been used for the effective therapeutics, their efficacy and significant side effects should be improved [12]. Because rare variants in Toll-like receptor 7 and sex-biased expression of the TLR7 gene influence severity of the patients’ symptoms [13,14], manipulation of antiviral receptors such as TLR7 could be an effective therapeutic target to synergize the cellular effect of current therapeutics. Our findings are clinically significant in that the direct ligand of TLR7, miRNA let-7b [15], is sequestered by CoVs’ Nsp9 protein in host cells to prevent antiviral responses. They also provide novelties for studying molecular mechanisms of how a viral protein utilizes cellular miRNA metabolism [16–19] to maintain its virulence and facilitate its transmission.

## Results

### Nsp9 is a microRNA-binding protein encoded by SARS-CoV-2 genome

**Figure 1.**
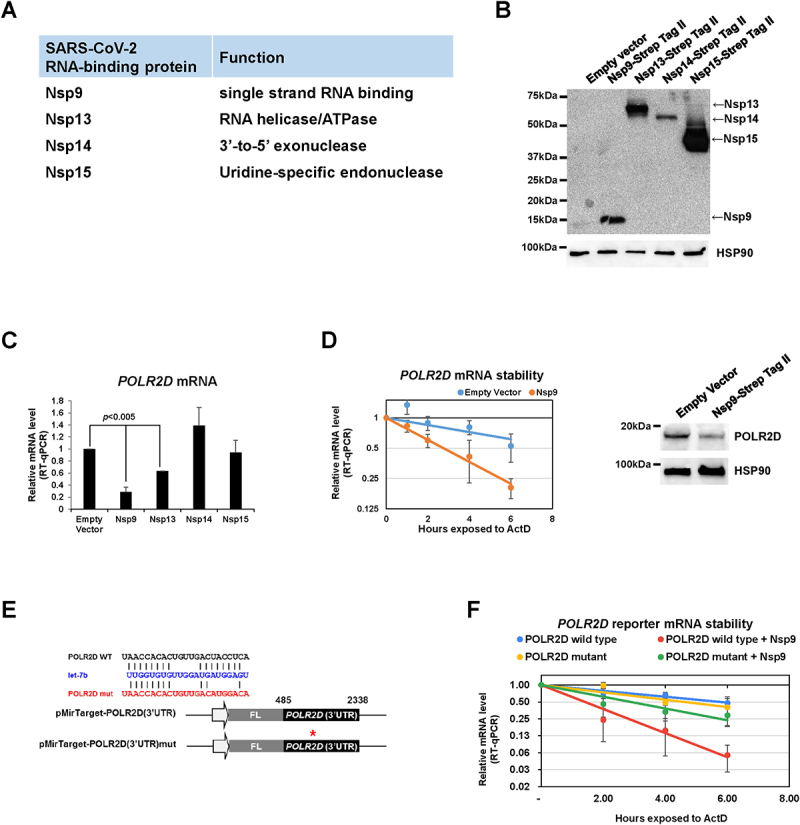
SARS-CoV-2 Nsp9 promotes let-7b-mediated *POLR2D* mRNA decay.

We initially examined the function of SARS-CoV-2 RNA-bidnding proteins (RBPs) such as Nsp9 (ssRNA-binding protein), Nsp13 (RNA helicase), Nsp14 (exoribonuclease), and Nsp15 (endoribonuclease) (Figure 1A) in host RNA metabolism. Human cell lysates (HeLa) ectopically expressed with Strep-tagged Nsp9, Nsp13, Nsp14, or Nsp15 (Figure 1B) were subjected to RT-qPCR for comparing the level of mRNA encoding POLR2D, a subunit of RNA polymerase II, plays a crucial role in RNA metabolism, encompassing transcription, RNA processing, RNA transport, and RNA stability. Consequently, POLR2D is a key player in the regulation of gene expression, significantly influencing various cellular processes [20]. Overexpression of Nsp9 decreased steady-state level of *POLR2D* mRNA (Figure 1C) as well as its stability (Figure 1D, ***left***). Consequently, the levels of POLR2D protein were significantly reduced due to the overexpression of Nsp9 (Figure 1D, ***right***). We observed that not only Nsp9, but also the overexpression of other Nsp proteins, particularly Nsp13, resulted in a significant decrease in *POLR2D* mRNA levels (Figure 1C). Since POLR2D is targeted by miRNA let-7b [18], we employed a reporter plasmid of firefly luciferase that includes the 3’ UTR of *POLR2D* mRNA, with and without the let-7b target site. Previous studies have demonstrated that these reporters accurately reflect the status of let-7b/Argonaute 2 (AGO2)-dependent *POLR2D* mRNA decay [18,21] (Figure 1E). Stability measurement of the reporter mRNAs revealed that overexpression of Nsp9 accelerates the decay of wild-type reporter mRNA but not the mutant defective in let-7b regulation (Figure 1F). These findings demonstrate that SARS-CoV-2 RBP, Nsp9 promotes let-7b-mediated decay of *POLR2D* mRNA.

(A) A list of SARS-CoV-2 RBPs. (B) Levels of Nsp9, Nsp13, Nsp14 and Nsp15 expressed in HeLa cells. (C, D) RT-qPCR level (C) or stability (D) of *POLR2D* mRNA (normalized with *GAPDH* mRNA or 18S ribosomal RNA respectively) using total RNAs purified from HeLa cells transfected with Strep-tag-Nsp9 plasmid or empty plasmid as a control for 48 h. For stability assay, cells were treated with 2.5 μg/μl Actinomycin D for the indicated times. Error bars represent the mean±SD of three independent experiments. *: *p* < 0.005. (E) A schematic of firefly reporter plasmids containing *POLR2D* 3’ UTR with or without mutation inhibiting let-7b-mediated decay. (F) RT-qPCR stability of *firefly* reporter mRNAs normalized with 18S ribosomal RNA using total RNAs purified from HeLa cells transfected with Strep-tag-Nsp9 plasmid or empty plasmid as a control for 48 h. Subsequently, cells were treated with 2.5 μg/μl Actinomycin D for the indicated times. Error bars represent the mean±SD of three independent experiments. *: *p* < 0.005.

**Table:** Primer sequences.

Name	Species	Sequence	Notes
let-7b	human/mouse	TGAGGTAGTAGGTTGTGTGGTT	
miR-130b	human/mouse	CAGTGCAATGATGAAAGGGCAT	
miR-21	human/mouse	TAGCTTATCAGACTGATGTTGA	
U6 RNA	human	CGCTTCGGCAGCACATATAC	
GAPDH F	human	AGCCACATCGCTCAGACAC	
GAPDH R	human	GCCCAATACGACCAAATCC	
POLR2D F	human	CAGCCCGTTTCAGTCGTTTC	
POLR2D R	human	CCAAACAGGCCAACTCAAACTT	
18S rRNA F	human	CGAACGTCTGCCCTATCAACTT	
18S rRNA R	human	ACCCGTGGTCACCATGGTA	
Luciferase (Firefly) F		ATGTACACGTTCGTCACATC	
Luciferase (Firefly) R		ACCTTTAGGCAGACCAGTAG	
IFNA1 F	human	ACCCACAGCCTGGATAACAG	
IFNA1 R	human	GAGGACAGAGATGGCTGGAG	
IFNB1 F	human	ACTGCCTCAAGGACAGGATG	
IFNB1 R	human	AGCCAGGAGGTTCTCAACAA	
TNF-α F	human	GAGGCCAAGCCCTGGTATG	
TNF-α R	human	CGGGCCGATTGATCTCAGC	
IL1B F	human	GGGCCTCAAGGAAAAGAATC	
IL1B R	human	TTCTGCTTGAGAGGTGCTGA	

**Figure 2.**
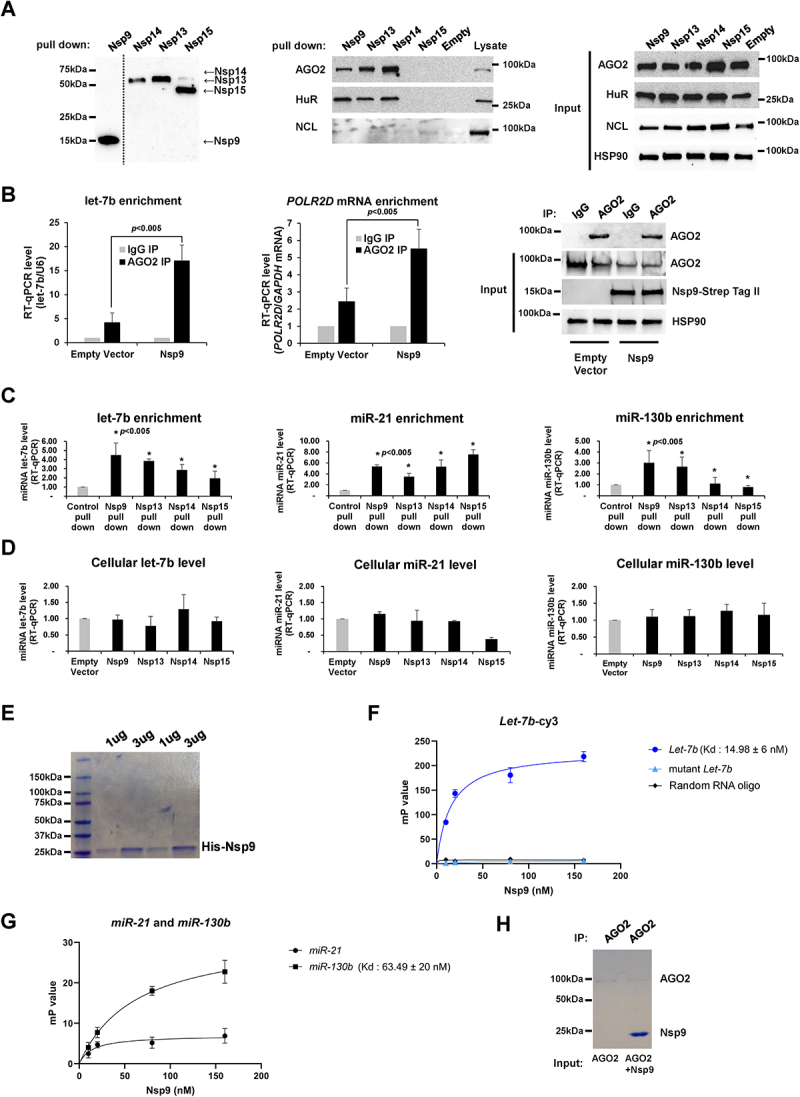
SARS-CoV-2 Nsp9 interacts with let-7b.

To elucidate the molecular mechanisms by which Nsp9 facilitates miRNA-mediated gene silencing, we conducted pull-down assays with Nsp proteins to assess their interaction with AGO2. The pull-down efficiency for each overexpressed Nsp protein was evaluated using Western blot analysis (Figure 2A, ***left***). We observed that Nsp9 is a part of a complex containing AGO2 in HeLa cell lysates after ectopic expression of Strep-tagged Nsp9. In addition, HuR, another component of the RNA-induced silencing complex (RISC) [22,23], has been shown in a previous study to interact with Nsp9, but not with NCL in HeLa cell lysates (Figure 2A, ***middle***). Therefore, Nsp9 May also be a component of RISC, similar to HuR. The protein expression levels of AGO2, HuR, and NCL proteins are similarly observed in cells overexpressing each of the Nsp proteins (Figure 2A, ***right***). Ectopic expression of Nsp9 enhanced enrichment of let-7b (Figure 2B, ***left***) and *POLR2D* mRNA (Figure 2B, ***middle***) on AGO2. Additionally, the pull-down efficiency of Ago2 in Nsp9-overexpressing cells with control was evaluated using Western blot analysis (Figure 2B, ***right***). Our molecular docking simulations of Nsp9 (pdb code; 6WXD) [19] and series of let-7b fragments indicate that the 5’-end region of let-7b possibly interacts with two β strands (β2 and β3) and a loop connecting them (Fig. S1A and S1B). Regarding the model, let-7b fits likely either an interface (Fig. S1C) or a crossover of Nsp9 dimer (Fig. S1D). We also observed that pull-down of Nsp9 with Strep-tag II [24] enriches let-7b (Figure 2C, ***left***) without changing steady state level of let-7b (Figure 2D, ***left***) as well as other miRNAs (Figure 2C,D, *middle and right*) possibly as a part of a complex with AGO2. Fluorescence Polarization of let-7b-Cy3 and recombinant Nsp9 (Figure 2E) revealed K*d* ~15 nM comparable to other miRNA-binding proteins (miRBPs). However, Nsp9 did not bind to random N20 RNA oligo (random-oligo) or the mutant let-7b-Cy3, which was designed with six substitutions within the 3’ end region to suppress its binding with other RBPs such as QKI [26]. This implies also the importance of the 3’ end sequences of miRNA in binding with Nsp9 (Figure 2F). Other miRNAs, such as miR-21 and miR-130b either do not bind to the Nsp9 protein (for miR-21) or bind with significantly weaker affinity compared to let-7b (for miR-130b), with a Kd of approximately 63 nM, respectively (Figure 2G). Although miR-21 and miR-130b were significantly enriched in the Nsp9 pull-down, as shown in (Figure 2C, ***middle and right***), we prioritized the investigation of the relationship between Nsp9 and let-7b due to the stronger binding observed, in contrast to the weaker associations of miR-21 and miR-130b with Nsp9. Furthermore, the use of recombinant Nsp9 in conjunction with AGO2 protein for pull-down assays clearly demonstrated a direct interaction between Nsp9 and AGO2 (Figure 2H). Our findings revealed that Nsp9 facilitates interaction of AGO2 and let-7b possibly by direct binding with let-7b to shut down expression of a subunit of RNA polymerase II, POLR2D.

(A) Western blot analysis of the pull-down efficiency of Nsp9, Nsp13, Nsp14, and Nsp15 overexpressed in HeLa cells (*left*). AGO2, HuR, and NCL from pull-down pellets using lysates from HeLa cells transfected with Nsp9, Nsp13, Nsp14, Nsp15, or empty plasmid for 48 h (*middle*). Total cell lysates were probed with antibodies against AGO2, HuR, NCL and HSP90 as well (*right*). The images are representative of at least three independent experiments. (B) RT-qPCR level of let-7b (normalized with *U6* RNA) (*left*) or *POLR2D* mRNA (normalized with *GAPDH* mRNA) (*middle*) using RNAs pulled down with AGO2 from lysates of HeLa cells transfected with Strep-tag-Nsp9 plasmid or empty plasmid as a control for 48 h. Western blot analysis of the pull-down efficiency of AGO2 and total cell lysates was probed with antibodies against AGO2, HuR, Nsp9-Strep Tag II, and HSP90, as shown (*right*). Antibody against normal mouse IgG was used as background control. Error bars represent the mean±SD of three independent experiments. *: *p* < 0.005. (C) RT-qPCR level of let-7b (*left*), miR-21 (*middle*), and miR-130b (*right*) (normalized with *U6* RNA) using RNAs pulled down with Nsp9, Nsp13, Nsp14, or Nsp15 from lysates of HeLa cells transfected with Strep-tag-Nsp plasmids or empty plasmid as a control for 48 h. Error bars represent the mean±SD of three independent experiments. *: *p* < 0.005. (D) RT-qPCR level let-7b (*left*), miR-21 (*middle*), and miR-130b (*right*) (normalized with *U6* RNA) using total RNAs purified from HeLa cells transfected with Strep-tag-Nsp9, Nsp13, Nsp14, Nsp15 plasmid or empty plasmid as a control for 48 h. Error bars represent the mean±SD of three independent experiments. *: *p* < 0.005. (E) Purification of recombinant His-Nsp9 and Coomassie blue staining of the protein. (F) Fluorescence Polarization of Nsp9 with let-7b-Cy3, mutant let-7b-Cy3, and N20 random RNA oligo. (G) Fluorescence Polarization of Nsp9 with miR-21 and miR-130b. (H) In vitro pull-down assays with AGO2 protein were conducted by incubating recombinant AGO2 protein alone or together with recombinant Nsp9 protein. Signals were detected using Coomassie Blue staining.

### Chemical shift assignments of Nsp9 and Nsp9-let-7b complex distinct from BMRB entry 50,622

**Figure 3.**
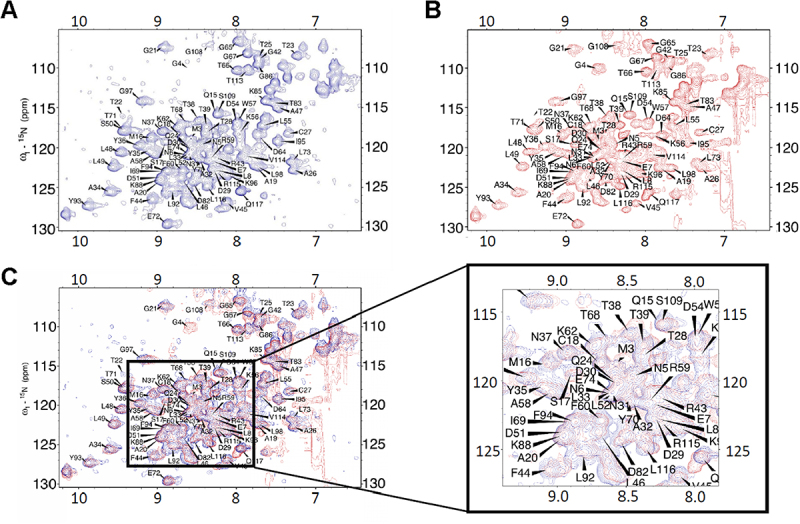
Nsp9 and let-7b interaction analysis via ^1^H, ^15^N-HSQC NMR spectrum.

In order to characterize the binding interface between Nsp9 and let-7b, we employed NMR perturbation studies to identify specific interaction sites by analysing 2D NMR spectra. The ^1^H, ^15^N-HSQC spectrum of Nsp9 (Figure 3A) and Nsp9/let-7b (Figure 3B) shows well-dispersed peaks, indicating that Nsp9 is properly folded in solution regardless of let-7b binding. The overlaying of these two spectra reveals many overlapping peaks, but some signals display chemical shift changes (Figure 3C), suggesting specific interactions upon binding.

(A) Spectrum of Nsp9 alone; (B) Spectrum of Nsp9 in complex with let-7b; (C) Overlay of two spectra, with a zoom-in view from 7.5 ppm to 9.5 ppm.

**Figure 4.**
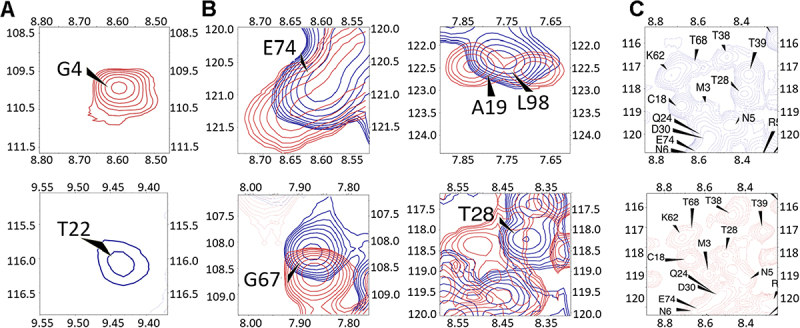
Observation of specific residues.

Specifically, the signal corresponding to the G4 residue, located at the N-terminus, is notably absent in the Nsp9 spectrum (Figure 3A). However, this signal reappeared in the spectrum in the presence of let-7b (Figures. 3B and 4A, **top**), indicating a direct binding site for let-7b. Conversely, the peak for the T22 residue, which was present in the spectrum of Nsp9 alone (Figure 3A), disappeared in the spectrum of the Nsp9/let-7b complex (Figures. 3B, and 4A, **bottom**), demonstrating another potential interaction site.

(A) G4 only presents in the Nsp9 with let-7b complex spectrum and T22 only presents in the Nsp9 spectrum; (B) Identification of significant signal changes in residues T28, G67, E74, A19, and L98; (C) More signal observation around M3 and N5 regions in Nsp9 with let-7b complex spectrum (Red) compare with Nsp9 spectrum (Blue)

Beyond these two specific changes, the other significant chemical shifts alterations were observed in residues T28, G67, and E74, as shown in Figure 4B. These observations demonstrated that the binding of let-7b and Nsp9 changed the chemical environment of residues, causing chemical shifts changes. These residues contributed to identifying the binding interface of Nsp9 with let-7b. Furthermore, the comparison of the two spectra revealed a pronounced transformation in the signals of residues A19 and L98 (Figure 4B). The initial sharp and intense signal in the Nsp9 spectrum was split into two broadened signals in the Nsp9/let-7b spectrum, which revealed that these two residues also constitute potential binding sites. Furthermore, the region nearby the N-terminal domain, particularly around M3 and N5 exhibited additional signals (Figure 4C), highlighting the involvement of N-terminal domain in the binding function of Nsp9 to let-7b.

### TALOS-N results insight on Nsp9 flexibility and rigidity

**Figure 5.**
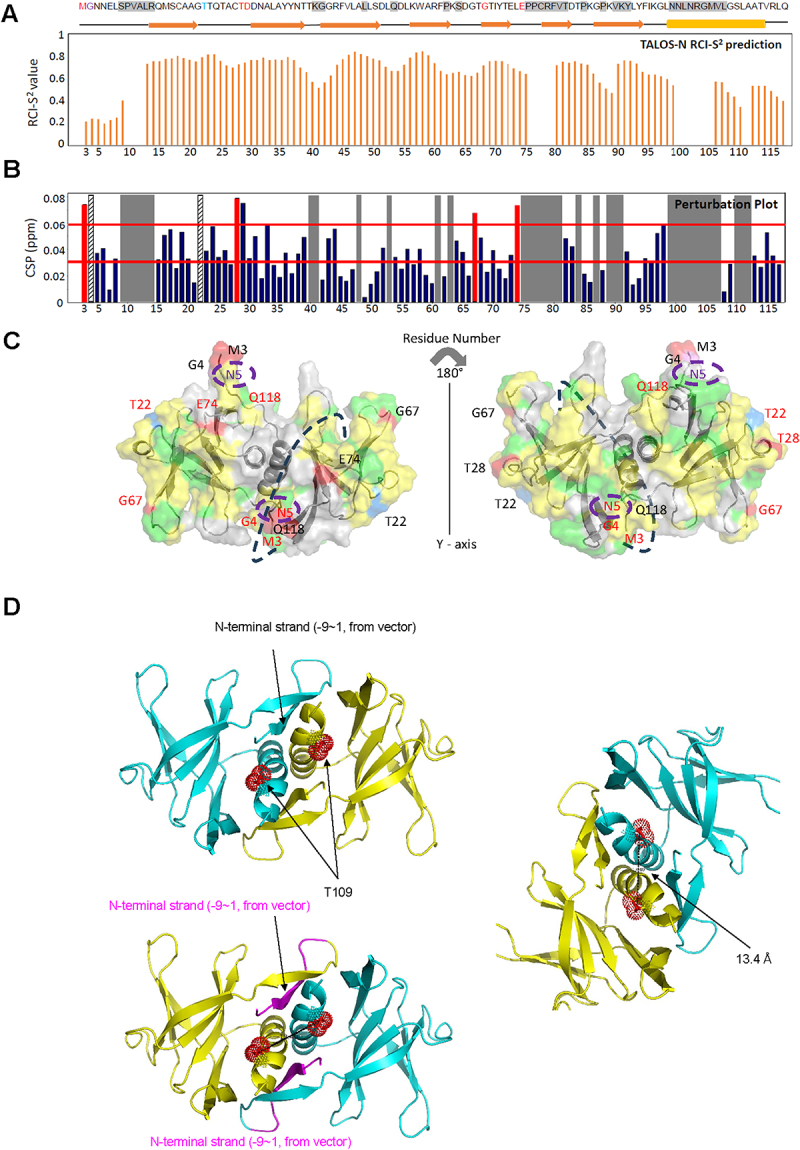
Structural analysis of Nsp9 upon let-7b interaction. (A) The RCI-S^2^ value of TALOS-N plot indicates the Nsp9 region’s flexibility (value near 0) and rigidity (value close to 1); (B) Chemical Shift Perturbation (CSP) illustrating the chemical shift differences between Nsp9 and Nsp9/let-7b complex, categorizing shifts into three regions, CSP > 0.06 ppm, CSP value between 0.03 ppm and 0.06 ppm, and CSP < 0.03 ppm. G4 and T22 are highlighted with stripes respectively, showcasing their unique spectral disappearances in the complex and isolated Nsp9; (C) Colour-coded dimer structure of Nsp9 based on CSP values: residues with CSPs > 0.06 ppm are depicted in red, while residues with CSPs between 0.03 ppm and 0.06 ppm are highlighted in yellow. G4 and T22 residues are uniquely coloured in violet and marine. N5, where the first N residue, is encircled by a dashed line. (D) Nsp9 phosphorylation structural models are presented, highlighting specific regions. Each Nsp9 dimer is depicted in cyan and yellow, with the N-terminal strand represented in magenta (-9 to 1, from the vector). The predicted phosphorylation site at residue T109 is marked in red within the alpha helices of each dimer. A distance measurement of 13.4 Å is indicated between the T109 phosphorylation sites of the dimer.

In order to further explore the role of N-terminal domain in the binding process of Nsp9 and let-7b, we also utilized the TALOS-N to analyse the flexibility of Nsp9 structure. According to the random coil index order parameter (RCI-S^2^) value calculations by TALOS-N for Nsp9 [1], the RCI-S^2^ values of residues in the N-terminal domain were closer to zero (Figure 5A). This result suggested greater flexibility in this region, so that the bonds would have a wider range of motion, which was beneficial for the binding process with let-7b. In addition, we also utilized perturbation plot to investigate the chemical shifts alternations of the Nsp9 residues resulting from the interaction with let-7b (Figure 5B).

### Identifying the significant signal changes through chemical shift perturbation (CSP) plots

The chemical shift perturbation (CSP) values were divided into three distinct categories, which represented different levels of structural alterations due to let-7b binding. Five shifts were greater than 0.06 ppm, thirty-eight shifts were ranging from 0.03 ppm to 0.06 ppm, and thirty-four shifts were below 0.03 ppm (Figure 5B). The higher CSP values indicated significant chemical shift changes at the residues, suggesting they were directly interacting with let-7b. Conversely, residues with CSP values lower than 0.03 ppm were likely peripheral to the binding areas and were not directly interacting with let-7b. The residues with CSPs value between 0.03 ppm and 0.06ppm exhibited moderate changes, and they might be located near the binding interface and potentially involved in the interaction with let-7b. The analysis of CSPs values not only helped us reveal the specific binding site of let-7b interaction, but also provided insights into the structural changes due to let-7b interaction.

### Exploring structural changes from let-7b interaction using PyMOL

To further understand the structural changes that occurred in Nsp9 upon let-7b interaction, we employed colour-coded 3D structural visualization methods by PyMOL. According to the CSPs values, residues M3, T28, G67, and E74 involved in significant chemical shift changes, were highlighted in red. Notably, these residues were primarily found in the random coil region. While residues that underwent moderate alternations, with CSPs values ranging from 0.03 ppm to 0.06 ppm, were coloured in yellow and mainly located in beta-sheet regions. Additionally, residue G4, which was only visible after the let-7b interaction, indicates a direct binding site of let-7b, was coloured in violet. Similarly, residue T22, which disappeared in the complex spectrum due to the presence of let-7b, was highlighted in marine (Figure 5C) and was also found in the random coil area. This observation revealed the binding interface of Nsp9 and let-7b was concentrated within random coil and beta-sheet regions, instead of the central alpha-helix of Nsp9, which contributed to the exploration of binding interface of Nsp9. Given that the phosphorylation of RBPs influences binding efficiency of RNAs [27–29], we conducted a structural analysis to identify potential phosphorylation sites on Nsp9. Our analysis indicated that the T109 may serve as a phosphorylation site in each dimer of Nsp9. Within Nsp9, the residues at positions G100 and G104 in GXXG motif, which are critical for Nsp9 dimerization [30], are in close proximity to T109. Furthermore, the distance between the phosphorylation sites in each dimer is approximately 13.4 Å (Figure 5D). This proximity may induce conformational changes in the protein, potentially modulating Nsp9 assembly and the RNA-binding efficiency of molecules such as let-7b due to electrostatic perturbations caused by the phosphate group [31,32].

## Nsp9 prevents TLR7-mediated inflammation by inhibiting the release of let-7b

**Figure 6.**
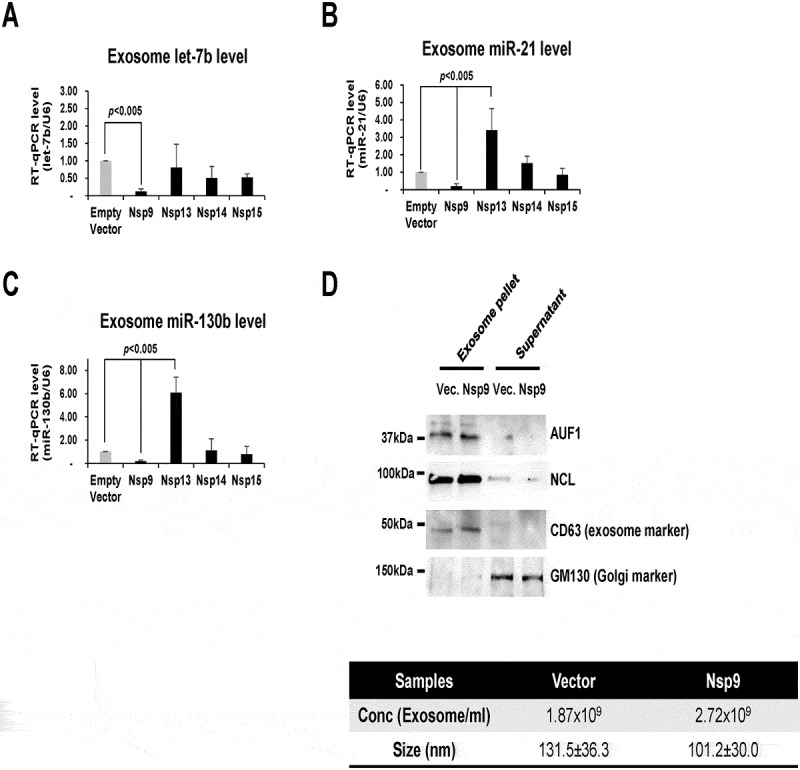
Nsp9 suppresses extracellular release of let-7b. (A-C) RT-qPCR levels of let-7b (*A*), miR-21 (*B*), and miR-130b (*C*) (normalized with *U6* RNA) using exosomal RNAs purified from HeLa cells transfected with Strep-tag-Nsp9, Nsp13, Nsp14, Nsp15 plasmid or empty plasmid as a control for 48 h. Error bars represent the mean±SD of three independent experiments. *: *p* < 0.005. (D) Western blot analysis of the exosome marker protein CD63, the Golgi marker protein GM130, as well as AUF1 and NCL in the exosome pellet and supernatant (*top*). The size of each exosome was measured using nanoparticle tracking analysis, as indicated in the table (*bottom*).

Since TLR7 is a receptor of let-7b [12], we hypothesized that SARS-CoV-2 proteins suppress activation of TLR7 by reducing the amount of its ligand, let-7b, in the extracellular space. Purification of extracellular vesicles (EVs or exosomes) from HeLa cells transfected with Nsp9 expression plasmid revealed that ectopic expression of Nsp9 highly reduced the amount of let-7b in exosomes (Figure 6A) without changing the cellular level of let-7b (Figure 2D). The abundance of miR-21 and miR-130b in EVs also changed to a similar degree as let-7b (Figure 6B,C). These observations suggest that either miR-21 or miR-130b may be also sequestered by Nsp9 within cells, despite their binding efficiency to Nsp9 being significantly lower than that of let-7b (Figure 2G). Additionally, we observed a less significant reduction in the levels of exosomal let-7b following the overexpression of Nsp13, Nsp14, and Nsp15. Our findings indicate that Nsp13 and Nsp14 interacted with AGO2 (Figure 2A, ***middle***), suggesting that they may share similar molecular functions with Nsp9. To ensure the purity of the exosomes, we analysed their size, which measured 131.5 ± 36.3 nm for the vector and 101.2 ± 30.0 nm for Nsp9 overexpression (Figure 6D, ***bottom***). Additionally, we evaluated the presence of CD63 [33], a well-established exosomal marker protein, alongside GM130 [34], a Golgi marker protein used as a negative control, through Western blot analysis (Figure 6D, ***top***). The decrease of exosomal let-7b suggests that active mechanisms sequester let-7b inside human cells. If this is the case, let-7b-binding proteins should be competing with SARS-CoV-2 RBPs. To identify RBPs directly binding with let-7b, we utilized human protein arrays immobilized with 354 RNA-binding domains. We incubated the array slide with let-7b, miR-21, and miR-130b, those are 5’-end labelled with Biotin (Figure S2A). In the RNA-binding domain array, we identified 75 RBPs directly interacting with let-7b, 47 with miR-21, and 62 with miR-130b (cut-off <1000 original intensity). There were 37 RBPs commonly interacting with 3 miRNAs, while 9 RBPs uniquely bound with let-7b exclusively (Figure S2B). To identify specific RBPs associated with the let-7 family of miRNAs, we integrated protein array data with additional let-7 miRNAs, including let-7a, let-7c, and let-7d along with let-7b. We observed that 59 RBPs bind all 4 miRNAs and 46 bind only let-7b, 7c, and 7d (Figure S3). Interestingly, NCL binds to all four let-7 family miRNAs, suggesting that the regulation of gene expression by these miRNAs may be concurrently mediated by NCL. Taken together, we successfully identified a number of potential novel miRBPs that interact with let-7b, miR-21, and miR-130b.

**Figure 7.**
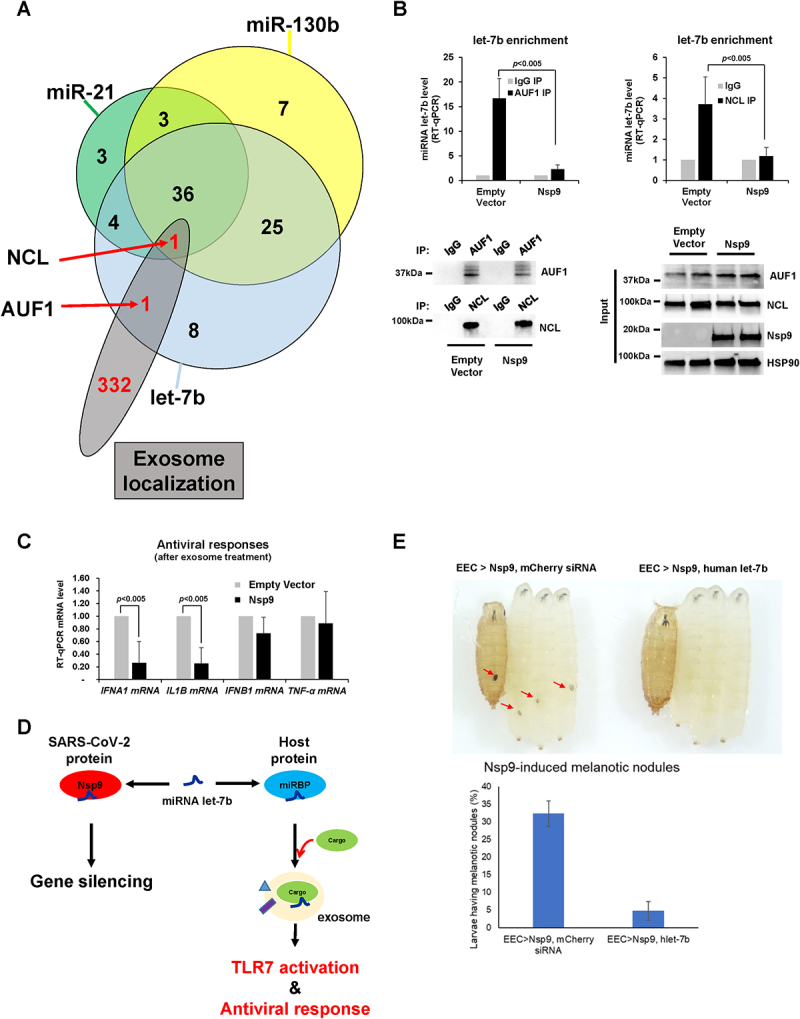
Nsp9 suppresses exosomal let-7b release and TLR7-mediated antiviral responses. (A) Comparison of proteins binding with let-7b identified from protein microarrays, and enriched in exosomes from Mass Spectrometry of exosomal lysates. Two common RBPs, AUF1 and NCL, were highlighted. (B) RT-qPCR level of let-7b (normalized with *U6* RNA) using RNAs pulled-down together with AUF1 (*top, left*) or NCL (*top, right*) from lysates of HeLa cells transfected with Strep-tag-Nsp9 plasmid or empty plasmid as a control for 48 h. Western blot analysis of the pull-down efficiency of AUF1 and NCL in Nsp9-overexpressing HeLa cells compared to control (*bottom, left*) and total cell lysates was probed with antibodies against AUF, NCL, Nsp9, and HSP90, as shown (*bottom, right*). Antibody against normal mouse IgG was used as background control. Error bars represent the mean±SD of three independent experiments. *: *p* < 0.005. (C) RT-qPCR level of TLR7-target mRNAs (normalized with *GAPDH* mRNA) using total RNAs purified from WI-38 cells exposed to exosomes for 2 h from HeLa cells transfected with Strep-tag-Nsp9 plasmid or empty plasmid as a control for 48 h. Error bars represent the mean±SD of three independent experiments. *: *p* < 0.005. (D) Proposed model of Nsp9 promoting let-7b-mediated gene silencing and suppressing extracellular let-7b release for antiviral responses. (E) Melanotic nodules in Nsp9-expressing larvae and pupae in the presence or absence of human let-7b (*top*). Quantification of larvae carrying melanotic nodules (*bottom*). More than 300 larvae were examined in three independent crosses. Error bars represent the mean±SD of three independent experiments. *p < 0.001.

If Nsp9 inhibits exosomal let-7b release, precise mechanisms should exist to assemble exosomes and to load let-7b for the accelerated release. While Nsp9 promotes interaction of AGO2 and let-7b, Nsp9 may sequester let-7b to the miRISC complex. If mammalian cells require cargo proteins to load let-7b into exosomes, such cargo proteins should interact directly with let-7b. To verify this hypothesis, we purified exosomes, analysed the exosomal proteins by Mass Spectrometry (MS) and identified 334 proteins (Figure 7A). Through a cross-comparison of 334 exosomal proteins identified by MS with 75 RBPs from the let-7b protein array (**Fig. S2B**), we identified AUF1 and NCL as the primary miRBPs of interest in exosomes (Figure 7A). Importantly, the protein abundance of AUF1 and NCL by Nsp9 remains unchanged across the nuclear, cytoplasmic (**Fig. S4A**), and exosomal fractions (Figure 6D, ***top***). Ectopic expression of Nsp9 reduced the amount of let-7b enriched in AUF1 or NCL (Figure 7B). The pull-down efficiency of AUF1 and NCL in IP (Figure 7B, ***bottom left***), along with the protein levels of Nsp9, AUF1, and NCL in Nsp9-overexpressing cells (Figure 7B, ***bottom right***), were validated through Western blot analysis. These results indicate that AUF1 and NCL could be the most important for loading let-7b to exosomes and their subsequent release.

Notably, miRNA let-7b is known to bind Toll-Like Receptor 7 (TLR7) on the surface of various cell types [15] such as macrophages, fibroblasts, hepatocytes, and neurons. When we transfected a plasmid expressing Nsp9 in HeLa cells, collected exosomes, and treated human lung cells with those exosomes, we observed decreased levels of cytokine mRNAs such as *IFNA1* and *IL1B* but not *IFNB1* and *TNF-α* (Figure 7C). These findings demonstrate that Nsp9 prevents let-7b release, leading to suppression of antiviral responses (Figure 7D).

Inspired by the generation of a comprehensive resource to study SARS-CoV-2 proteins in *Drosophila* [35], we established transgenic *Drosophila* lines expressing SARS-CoV-2 Nsp9 and observed its effects across different tissue types. Interestingly, a small subset of larvae presented with melanotic nodules, indicative of haemocyte activation and an innate immune response [36]. Further investigations identified the gut’s enteroendocrine (EEC) cells as the source of this immune response. When Nsp9 was specifically expressed in EEC cells using the split-GAL4 system, 32% of larvae exhibited melanotic nodules (Figure 7E, ***top left and bottom of Nsp9, mCherry siRNA***). Interestingly, co-expression of human let-7b with Nsp9 in EEC cells significantly reduced the formation of melanotic nodules (Figure 7E, ***top right and bottom of Nsp9, human let-7b***), suggesting that human let-7b suppresses the Nsp9-induced innate immune response in *Drosophila*.

Taken together our findings provide critical new insights into fundamental molecular mechanisms and post-transcriptional regulation that mediate antiviral responses against SARS-CoV-2 infection. Moreover, these studies will elucidate pathways that have direct clinical relevance by targeting inflammatory receptors in the lung. Thus, the work directly supports our long-term objective to develop new therapeutics that target an RNA ligand and its counterpart receptor in COVID-19.

## Discussion

### Nsp9 in miRNA-mediated gene silencing

Our study highlights the role of the SARS-CoV-2 RBP Nsp9 in regulating host gene expression through a miRNA-mediated mechanism. Among the various Nsps encoded by the SARS-CoV-2 genome, Nsp9 has been identified as a single-stranded RBP [37]. Nsp9 overexpression leads to a marked decrease in the stability and steady-state levels of *POLR2D* mRNA, a critical subunit of RNA polymerase II (Figure 1C,D). Since eukaryotic RNA polymerase II is essential for the transcription of most cellular mRNA, its downregulation would have significant impacts on cellular gene expression [38,39]. Additionally, the destabilization of *POLR2D* mRNA by Nsp9 requires the presence of let-7b seed sequence, and Nsp9 enhances the association of let-7b with AGO2, a key component of the RNA-induced silencing complex (Figure 2). The enhanced enrichment of let-7b on AGO2 in the presence of Nsp9 suggests that Nsp9 may facilitate or stabilize the interaction between AGO2 and miRNAs. Several cellular proteins, such as GW182 and DDX6, are known to interact with AGO2 and play roles in miRNA-mediated gene silencing mechanisms [40,41], thereby enhancing miRNA-mediated suppression of mRNA translation and acceleration of mRNA deadenylation. Moreover, our molecular docking simulations and biochemical assays support the hypothesis that Nsp9 directly interacts with the let-7b, particularly at the 5’-end region (Fig. S1A and S1B), which is crucial for target mRNA recognition. This interaction potentially increases the efficiency with which miRNAs are loaded onto AGO2, enhancing their gene-silencing capabilities. While it is currently unclear which other miRNAs may be affected by Nsp9 overexpression, it is possible that other members of the let-7 family, as well as miR-21 and miR-155, could be influenced due to their significant roles in gene regulation and immune response modulation, where miR-21 is known to regulate tumour suppressor genes and contribute to cancer progression, and miR-155 is involved in inflammatory responses and autoimmune diseases [42,43]. In the future, our Nsp90 CLIP-seq (Cross-Linking Immunoprecipitation followed by sequencing) data will deliver a comprehensive overview of the regulation of host RNA metabolism, presenting a transcriptome-wide map of RNA targets for Nsp9 and elucidating precise regulatory mechanisms.

While our findings suggest that Nsp9 influences the miRNA-mediated gene silencing pathway, it raises the question of whether SARS-CoV-2 infection promotes miRNA-mediated gene silencing as a broader strategy. Previous studies have shown that viruses often manipulate host miRNA pathways to enhance their replication and evade immune responses. For example, certain herpesviruses and polyomaviruses encode their own miRNAs to regulate host and viral genes [44,45]. This raises the possibility that SARS-CoV-2 might similarly exploit miRNA pathways. To date, there is limited evidence to suggest that SARS-CoV-2 encodes its own miRNAs [46,47]. However, given the diverse strategies viruses use to interact with host cells, it is plausible that SARS-CoV-2 could either encode viral miRNAs or modulate host miRNA expression to benefit its replication and survival. Recent bioinformatics analyses and deep sequencing efforts have begun to uncover small RNA profiles in SARS-CoV-2-infected cells, hinting at potential viral and host miRNA interactions [48]. Therefore, these findings suggest that Nsp9 influences the miRNA-mediated gene silencing pathway, highlighting its potential role in modulating host cell functions during SARS-CoV-2 infection. Further research is needed to explore whether SARS-CoV-2 encodes its own miRNAs and to elucidate the broader implications of viral manipulation of the miRNA machinery.

### Structure of let-7b with canonical RNA-binding proteins compared to Nsp9

Based on the observations of ^1^H, ^15^N-HSQC NMR spectrum and perturbation plot, the presence of let-7b led to structural changes in Nsp9 (Figure 3). Specifically, the presence of G4 and absence of T22 in the Nsp9/let-7b complex spectrum were directly caused by let-7b binding (Figure 4A). Additionally, 5 residues underwent significant chemical shift changes, while 38 residues showed moderate structural alterations (Figure 5B). These observations suggested that let-7b binding could alter the chemical environment surrounding residues, and these changes could also be used to identify the binding interface between Nsp9 and let-7b. Moreover, the region near residues M3 and N5, which was located at the N-terminus of Nsp9, displayed more signals after the presence of let-7b, suggesting the potential role of the N-terminal domain in the binding process. Integrating this result with the RCI-S^2^ plot, the residues in this area also exhibited higher flexibility, where the RCI-S^2^ values were closer to 0 (Figure 5A). This indicated that the N-terminal domain played a crucial role in the binding interaction between Nsp9 and let-7b.

The analysis of the 3D structure of the Nsp9 dimer contributed to revealing the binding interface between Nsp9 and let-7b (Fig. S1D). Residues T28, G67, and E74, which exhibit significant chemical shift changes due to let-7b binding, were primarily located within the random coil regions (Figure 4B). According to the essential characteristics of random coil, it would display higher flexibility, which could facilitate the interaction with let-7b. Conversely, the residues showing moderate changes, with CSP values ranging from 0.03 ppm to 0.06 ppm, were mainly found in the beta-sheet regions (Figure 5B). The beta-sheets might provide a substantial interface area that is conducive to let-7b binding.

The colour-coding analysis of the Nsp9 dimer structure showed that the interaction between let-7b and Nsp9 occurred primarily in the random coil and beta-sheet regions (Figure 5B, S1D). The intrinsic properties of these regions contributed to their critical role in the binding interface. Based on the observation of the binding interface, let-7b originated from the beta-sheet regions, and extended into the peripheral random coil areas, rather than the centrally located alpha-helix of Nsp9 (Figure 5).

Structural analysis identifies T109 on Nsp9 as a potential phosphorylation site (Figure 5D). Phosphorylation of RNA-binding proteins (RBPs) introduces a phosphate group, altering charge, structure, and interactions [49]. This modification can either enhance or reduce RNA-binding efficiency, depending on the phosphorylation site and protein structure [27–29,49]. Phosphorylation of T109 on Nsp9 introduces a negative charge, may alter the electrostatic pertubation and causing conformational changes. These changes may impact the stability or orientation of the dimer interface by affecting closed residue of Nsp9 between T109 and the G100,G104 residues in the GXXXG motif, which are critical for dimerization and essential for Nsp9’s structural stability and RNA-binding efficiency [30] of such as let-7b. However, the precise binding pathway of let-7b has not yet been determined, and further research and verification is needed. Although further experimental validation is needed to fully understand the binding process between Nsp9 and let-7b, this detailed mapping highlighted specific regions contributing to the interaction of let-7b.

### Nsp9 in TLR7-mediated inflammation

This study also highlights the critical role of Nsp9 in modulating the human innate immune response to SARS-CoV-2 infection. SARS-CoV-2 transcripts contain 5’-m^7^GpppN CAP, protecting the viral RNAs from decapping and facilitating the production of viral proteins [7,50–52]. Nsp9 cooperates with the Nsp12 NiRAN domain during capping, inducing RNAylation forming a covalent RNA-GDP intermediate. This process facilitates the transfer of RNA to GDP leading to the formation of the essential core CAP structure GpppA-RNA. This allows the virus to escape host immune surveillance because its degradation relies on the detection of cap structures distinct from host ones [7,53].

Our previous studies demonstrated that a subset of mature miRNAs directly bind with canonical RBPs, accelerating miRNA loading to AGO2, and then facilitates gene silencing [18]. Here we have demonstrated that Nsp9 enhances let-7b binding to AGO2 (Figure 2B) and accelerates decay of mRNA encoding RNA polymerase II subunit D, POLR2D (Figure 2). By highlighting let-7b role as an endogenous ligand for TLR7 in innate immunity [15], this study elucidates how Nsp9 suppresses immune activation by impeding let-7b-TLR7 interaction (Figures 2A and Figure 6A). Furthermore, we also explored TLR7-mediated signalling pathways, crucial for the type I interferon (IFN) response (INF alpha (α) and beta (β)) against viral infections [54]. However, a clinical study in SARS-CoV-2 patients demonstrated reduced INF-α production revealing lack of the type I INF response [55] and genetic variation in the TLR7 gene has been linked to severity of SARS-CoV-2 infection, underscoring the importance of TLR7 in mounting an effective antiviral response [56]. Our experimental results further support these clinical findings, demonstrating decreased levels of IFN α mRNA in conditions where Nsp9 is expressed (Figure 7C), suggesting Nsp9’s contribution to dampening the type I interferon response. Taken together, these findings suggest that Nsp9 is reducing miRNA let-7b availability to TLR7 dampening the type I interferon response and suppressing the antiviral response [57]. Targeting the availability of let-7b to TLR7 may hold a promising therapeutic strategy against SARS-CoV-2, providing potential avenues for intervention in combating the virus’s sophisticated immune evasion tactics.

### Genetic interactions between Nsp9 and let-7b in *Drosophila*

Our research investigated the roles of SARS-CoV-2 Nsp9 in *Drosophila*. Previous studies have indicated that Nsp9 expression in the wings leads to minor defects in wing veins, highlighting a genetic interaction with signalling pathways essential for wing development [35]. Given the significant symptoms associated with SARS-CoV-2 infection in the brain and gut, we employed Elav-GAL4 to drive pan-neuronal expression of Nsp9 and observe any phenotypes [58]. Consistent with earlier findings [58], we did not observe notable neurological abnormalities. However, we unexpectedly detected melanotic masses, or nodules, in a small subset of larvae, indicating the activation of haemocytes and innate immune responses [36]. This prompted us further investigation, revealing that the immune response was initiated in the gut’s EEC cells. Specifically, expressing Nsp9 in EEC cells using the split-GAL4 system resulted in 32% of larvae developing melanotic nodules. Intriguingly, co-expression of human let-7b with Nsp9 in EEC cells significantly reduced the formation of these nodules, suggesting that human let-7b effectively suppresses the Nsp9-induced innate immune response in *Drosophila* (Figure 7E). These observations underscore the utility of *Drosophila* as a robust *in vivo* model for studying SARS-CoV-2-induced immune responses, particularly those related to blood cell differentiation and aggregation. Our findings have broader implications for understanding the interactions between viral proteins and host immune pathways. The ability of human let-7b to modulate Nsp9-induced immune responses indicates potential regulatory mechanisms that could inform therapeutic strategies. Future research should focus on elucidating the molecular mechanisms underlying these interactions and exploring the applicability of this model to other viral proteins and their effects on host immunity.

## Method details

### Human cell culture

HeLa cells (**ATCC® CCL-2™**) and WI-38 cells (**Coriell Institute**) were cultured in DMEM supplemented with 10% FBS and penicillin/streptomycin. SARS-CoV-2 plasmids were obtained from Addgene and transfected to HeLa cells using polyethyleneimine.

### Subcellular fractionation

Cytosolic and nuclear fractions were isolated as previously described [59]. In brief, cells were lysed in a buffer containing 10 mM Tris-HCl (pH 7.4), 100 mM NaCl, 2.5 mM MgCl2, and 40 μg/ml digitonin for 10 min. The resulting lysates were then centrifuged at 2,060 g for 10 min at 4°C, and the supernatant was collected as the cytosolic fraction. The pellets were washed and incubated with RIPA buffer at 4°C for an additional 10 min. After centrifugation at 21,000 g for 10 min at 4°C, the nuclear fraction was obtained.

### Exosome preparation

Exosome isolation was performed as previously described [60,61]. Briefly, the conditioned medium containing extracellular vesicles from cell culture was collected and centrifuged at 2,000 × g for 15 min, then at 10,000 × g for 30 min. The resulting post-microvesicle supernatant was spun at 100,000 × g for 2 h. The resulting exosome pellets were then resuspended with PBS and re-spun at 100,000 × g for 2 h. The resulting final exosome pellets were suspended in PBS for further analysis.

### SDS gel electrophoresis and Western blot analysis

Whole‐cell lysates, prepared in radioimmunoprecipitation assay (RIPA) buffer, were separated by sodium dodecyl sulfate‐polyacrylamide gel electrophoresis (SDS‐PAGE) and transferred onto nitrocellulose membranes (Invitrogen iBlot Stack). Immunoblots were performed using antibodies against AUF1 (1:2000, Millipore 07–260), HuR (1:2000, SCBT sc-5261), AGO2 (1:2000, Abcam ab57113), NCL (1:2000, Abcam ab27758), Histone H2B (1:2000, SCBT sc -515,808), POLR2D (1:2000, Abcam ab229488), HSP90 (1:2000, SCBT sc -515,081), CD63 (1:2000, Sigma SAB5701163), GM130 (1:2000, Sigma SAB5700801), Strep-tag (1:1000, Qiagen). Horse Radish Peroxidase (HRP)‐conjugated secondary antibodies were purchased from GE Healthcare.

### RNA immunoprecipitation

RNA immunoprecipitation (RIP) analysis from whole cell extracts was performed as described previously [18,29]. Briefly, cells were lysed in buffer containing 20 mM Tris-HCl (pH7.5), 100 mM KCl, 5 mM MgCl_2_, 0.5% NP-40 and cleared by centrifugation. The lysates were incubated with protein A-Sepharose beads coated with antibodies against AGO2, HuR, NCL or AUF1 with control IgG (SCBT) at 4°C for 1 h. For pull down of Strep-tag Nsp9, Nsp13, Nsp14, or Nsp15, streptavidin beads (Pierce) were incubated with lysates from HeLa cells transfected with Nsp9, Nsp13, Nsp14, or Nsp15 plasmid for 3 h. After the beads were washed four times with NT2 buffer [50 mM Tris-HCl (pH 7.5), 150 mM NaCl, 1 mM MgCl_2_, 0.05% NP-40], the immunoprecipitates were treated with 20 units of RNase-free DNase I at 37°C for 15 min and proteinase K (0.5 mg/ml) in 0.1% SDS at 55°C for 15 min to remove DNAs and proteins, respectively. The RNAs isolated from the IPs by acidic phenol extraction were then subjected to RT-qPCR using the primers listed in Table S1. The RIP results were normalized to *GAPDH* mRNA.

### Real-time RT-qPCR

Total RNA was isolated with Trizol reagent (Invitrogen) and reverse-transcribed with the Maxima reverse transcriptase (Fermentas). Expression of specific mRNAs was determined with iCycler (Bio-Rad) using the KAPA SYBR FAST qPCR kit (Kapa Biosystems). After reverse transcription, cDNAs were quantitated by qPCR with mRNA-specific primers or with primers to detect the control transcript ***GAPDH***. All the sources of qPCR primers used are listed in Table S1. miRNA quantitation was conducted after RNA extraction using polyadenylation (QuantiMiR kit, #RA420A, System Biosciences) and hybridization with oligo-dT adaptors. Following reverse transcription, cDNAs were quantified by qPCR, utilizing either miRNA-specific primers or primers designed to detect the control transcript U6 snRNA, along with a universal primer. U6 snRNA served as the normalization standard for exosomal miRNA, as previously described [26].

### Recombinant protein purification and in vitro pull-down

Recombinant proteins were purified as previously described [21]. Recombinant AGO2 was purchased from SinoBio (Cat # 11079-H07B). *E. coli* BL21 (DE3) cells transformed with plasmids encoding Nsp9 were cultured in LB medium at 37°C to an OD600 of 0.6–0.8. Protein expression was induced with 0.5 mM IPTG for 3 h. Bacterial pellets were sonicated in ice-cold lysis buffer (20 mM Tris-HCl pH 7.4, 300 mM NaCl, 0.1% NP-40, 20 mM EDTA, 10% glycerol) and subsequently centrifuged. The cell debris was removed by centrifugation at 18,000 rpm for 2 h, and the protein was captured using Ni-NTA (Qiagen) affinity chromatography, followed by further purification with Superdex S200 (GE Healthcare) equilibrated in a solution containing 50 mM Tris-HCl, 300 mM NaCl, and 1 mM DTT. The size and concentration of the purified protein were confirmed using the Bradford protein assay and Coomassie blue staining. Recombinant AGO2 or AGO2 with Nsp9 were mixed, subjected to pull-down using an AGO2 antibody, and analysed by Coomassie blue staining.

### Docking simulation of Nsp9 dimer and let-7b fragments

The crystal structure of Nsp9 was obtained from protein data bank (PDB code; 6WXD). All the molecular dockings with a Nsp9 dimer and 9 sets of 6-nucleotides let-7b fragments were carried out by PyRx Virtual Screening Tool Autodock Vina Version 0.9.7, at every RNA fragment for 5 times per model, followed by a determination of the minimum docking energy reflected among 15 calculated values [62]. For running the software, the PDB file was converted to PDBQT format via OpenBabelGUI program [63]. The docking models were presented by PyMOL software (The PyMOL Molecular Graphics System, Version 2.0 Schrödinger, LLC.)

### Fluorescence polarization assay

To confirm the RNA-binding affinity of Nsp9, recombinant His-Nsp9 was combined with 100 nM Cy3-labelled let-7b (5’-/phospho/UGAGGUAGUAGGUUG**UGUGGU**U-Cy3–3‘), 100 nM mutant Cy3-labelled let-7b (5‘-/phospho/-UGAGGUAGUAGGUUG**CCAUUA**U-Cy3–3‘), 100 nM Cy3-labelled 20-mer random oligo of RNA (N20, 5‘-/phospho/-UGAGGUAGUAGGUUGCCAUUAU-Cy3–3‘), 100 nM Cy3-labelled miR-21 (5‘-/phospho/-UAGCUUAUCAGACUGAUGUUGA-Cy3–3‘), and 100 nM Cy3-labelled miR-130b (5‘-/phospho/-CAGUGCAAUGAUGAAAGGGCAU-Cy3–3’) in reaction buffer (20 mM HEPES pH 7.0, 150 mM NaCl, 2 mM MgCl_2_, 10 mM DTT and 1 U/ul RiboLock RNase inhibitor), respectively. Solution of protein and RNA were transferred to 96-well non-binding black plate (Corning) and incubated for 5 min at room temperature. Fluorescence polarization was measured using a Spectramax iD5 microplate reader (Molecular Device) with 530 nm excitation and 570 nm emission wavelengths. Millipolarization units (mP) were plotted against Nsp9 concentrations, and fitting curve was performed by nonlinear regression using a one-site binding model in GraphPad Prism.

### Human protein microarray analysis

The Invitrogen Ultimate 16,813 ORF collection, subcloned into a yeast expression vector that allows for galactose-dependent overexpression of N-terminal GST- and 6x-His-tagged recombinant proteins, was spotted onto Fullmoon slides (Fullmoon Biosystem) using the NanoPrint LM210 system (ArrayIT) as previously described [25,64]. The quality of these protein microarray chips, along with control proteins (such as IgG, GST, and histones) were assayed using a randomly selected slide from the same batch, and at least 89% of the proteins were expressed correctly.

To identify miRNA-binding proteins, the protein microarray chips were equilibrated and washed three times for 15 min with RNase-free PBS pH 7.0 with 2 U/μL RNase inhibitor, SUPERase-In^TM^ (Thermo Scientific). The proteins on the chips were blocked with filtered 3% high-quality IgG-/protease-free BSA (Jackson Immuno Research Laboratory Inc.) for 1 h at room temperature, then washed three times with RNase-free PBS buffer (filtered RNase-free PBS pH 7.0 with 1 U/μL RNase inhibitor). The protein microarray chips were incubated with internally Cy5- or DY647-labelled let-7b, miR-21, and miR-130b (100 ng) for 1 h, wrapped in aluminium foil on a shaking platform at 50 rpm. The chips were washed five more times with RNase-free PBS buffer for 10 min.

For detection of all possible proteins, the chips were then incubated with rabbit anti-GST antibody (Millipore) at 1:5,000 for 1 h at room temperature, washed three times for 15 min in RNase-free PBS buffer, and incubated with 1:1,000 Cy3 goat anti-rabbit IgG antibody (Invitrogen) in RNase-free PBS buffer for 20 min at room temperature. The final washing comprised three ×15 min in RNase-free PBS buffer, and rinsed once in double-distilled H_2_O. Protein microarray chips were dried via centrifugation at 200 g for 2 min using a 50 ml conical tube.

The chips were scanned with GenePix 4000B (Axon Instruments) for quantification and statistical analysis. Fluorescence from bound Cy5/DY647-labelled let-7b, miR-21, and miR-130b was measured at 635 nm, and from Cy3-labelled anti-GST at 532 nm. Signal intensity values for each spot were obtained through scanning, to find the ratio of foreground to background signals, and normalized with GST signal intensity. The mean and standard deviation of signal intensity of all proteins on the chip were calculated using R package, and *p* ≤ 0.05 was considered statistically significant.

### Gene ontology analysis

The list of 60 identified let-7b-binding proteins was submitted to the online DAVID functional annotation resource (v6.8) [65,66], where the overrepresented proteins were identified and compared with the human proteome database.

### NMR experiments

The pET-28a-3C protease recombinant SARS-CoV-2 nsp9 gene was cultured in M9 labelled media for 15N, consisting of 1 g/L ^15^NH_4_Cl, 3 g/L D-Glucose, 0.5 g/L ^15^N ISOGRO, and 100 mg/L kanamycin at 37°C. IPTG induction occurred at OD 600 nm of 0.8, followed by protein expression at 37°C for 6 h. Cell lysis was performed using sonication after resuspending the cell pellet in lysis buffer (20 mM HEPES, 20 mM Imidazole, 0.5 mM TCEP, pH 7.0). The supernatant was filtered using a 0.2 μm vacuum filter before loading to a His-Trap column, eluted with wash buffer (20 mM HEPES, 100 mM Imidazole, 0.5 mM TCEP, pH 7.0) and elution buffer (20 mM HEPES, 500 mM Imidazole, 0.5 mM TCEP, pH 7.0), and dialysed overnight in dialysis buffer (20 mM HEPES, 150 mM NaCl, pH 7.0). Cleavage with 3C protease occurred for 12 h at 4°C with 2 mM DTT. 3C protease and epitope tag were removed via a second His-Trap column purification. The protein was further purified with the AKTA START fast protein liquid chromatography (FPLC) using the 3C protease buffer (20 mM HEPES, 15 mM NaCl, 0.5 mM TCEP, PH 7.0). The final NMR sample, approximately 0.5 mM in protein concentration, was prepared by concentrating it to 750 µL and supplementing with 0.02% sodium azide and 10% deuterium oxide (D_2_O) to ensure stability and effective deuterium incorporation. 2D ^1^H,^15^N-HSQC experiment of ^15^*N*-labelled Nsp9 was conducted at 298 K using a 400 MHz Bruker spectrometer, employing parameters such as 128 points in the frequency domain, 512 points in the time domain, 16 dummy scans, and 1024 total scans to ensure optimal spectral acquisition and resolution. Following the NMR experiment of Nsp9, the Nsp9/let-7b complex sample was prepared by recovering the Nsp9 sample from the NMR tube, then concentrating and adding 4 mM let-7b with 5’ phosphoryl group and 3’ hydroxyl group to achieve a final volume of 750 µL. The 4 mM let-7b was slowly added at a 1:10 molar ratio to the protein concentration to ensure optimal interaction. The Nsp9/let-7b complex NMR experiment was conducted using the same NMR parameters and experimental conditions as the initial Nsp9 study.

### NMR data analysis

Acquired time-domain NMR data were processed using NMRPipe [67] to attain high-quality frequency-domain NMR data by employing window apodization, zero-filling and phase correction. Processed spectra were analysed using the POKY suite (BUILD 112,723) [68]. Nsp9 backbone chemical shifts deposited in the BMRB by Pastore group (entry number 50,622) were imported into POKY via the BMRB entry download button within the Resonances tab in POKY [69]. Then, we initially generated assigned peaks on the ^1^H,^15^N-HSQC of Nsp9 utilizing the Transfer-and-Simulate tool (two-letter-code ‘ta’) [70]. We verified and adjusted assigned peaks on ^1^H,^15^N-HSQC of Nsp9 with the help of the peak list (two-letter-code ‘lt’) and REDEN (two-letter-code ‘re’) [71]. Subsequently, all adjusted assignments were transferred onto the Nsp9/let-7b complex spectrum by copy and paste shortcuts (control-a, control-c and control-p). We also verified and adjusted assigned peaks on ^1^H,^15^N-HSQC of Nsp9/let-7b complex.

To quantify the chemical shift changes of Nsp9 induced by let-7b binding, the Perturbation Plot tool was used, which is also available in the POKY suite (two-letter code ‘np’). This involved the calculation of chemical shift perturbation (CSP) values using the equation:

$$\mathrm{Chemical}\;\mathrm{Shift}\;\mathrm{Perturbation}\left(\mathrm{ppm}\right)=\sqrt{{\left(\Delta \delta^{1}H\right)}^{2}+{\left(\Delta \delta^{15}N/5\right)}^{2}}$$

where $\Delta \delta^{1}H$ and $\Delta \delta^{15}N$ represent the alterations in chemical shifts of hydrogen and nitrogen nuclei, respectively, upon let-7b binding.

The 6WXD structure from the Protein Data Bank was utilized as the reference configuration for the Nsp9 protein dimer, which notably lacks G4 residues at the N-termini of both chains A and chain B, and N5 residue at the N-terminus of chain B. To enhance the understanding of the Nsp9 protein’s binding affinity with let-7b, PDBFixer was employed to introduce the missing G4 and N5 residues to the N-termini in the Nsp9 model to better identify the potential binding interface with let-7b [72]. PyMOL was employed to visualize the structural alterations within the 3D structure of the Nsp9 dimer, with CSP values guiding the colour coding of regions that exhibit significant shifts due to let-7b presence. By utilizing the ‘Surface’ feature in PyMOL, a comprehensive solid surface view of the Nsp9 structure was obtained. The transparency was adjusted to 60% to enhance visualization clarity. TALOS-N was employed to assess the flexibility of a region within Nsp9 [73].

### Transgenic *Drosophila* generation, maintenance, and phenotype analysis

To express Nsp9 in *Drosophila*, we synthesized and cloned Nsp9 with the additional Kozak sequence and ATG in pUASTattb (Genscript Inc) and inserted it into attp2 (a third chromosome landing site) with Bestgene Inc. To express human let-7b, we used a *Drosophila* let-7 precursor backbone by replacing the mature *Drosophila* let-7 sequence with the human let-7b sequence. It was synthesized and cloned in pUASTattb by Genscript and inserted in attp40 (a second chromosome landing site) by Bestgene. Transgenic fly and GAL4 lines were maintained at 25°C with a diurnal 12/12 (day/night) cycle with the standard Bloomington food. Melanotic nodule formation was visually inspected using wandering third instar larvae of corresponding genotypes under the standard stereoscope setting. Unless otherwise mentioned, three independent crosses were used to determine the frequency of melanotic nodule formation.

### Quantification and statistical analysis

Data are expressed as the mean ± SD of the values from at least three independent experiments performed, as indicated in the corresponding figures legends. The numbers of biological replicates, and what they represent, are indicated in each figure legend. Two-tailed Student’s *t* tests were used for single comparison. *p* values below 0.05 were considered statistically significant.

## Supplementary Material

NSP9 (Figures) Final_edit_Page_09.tif

NSP9 (Figures) Final_edit_Page_10.tif

NSP9 (Figures) Final_edit_Page_11.tif

NSP9 (Figures) Final_edit_Page_08.tif

## Data Availability

NMR data associated with study is available in Biological Magnetic Resonance Bank under the entry number of **52552** (^1^H,^15^N-HSQC chemical shift assignment of SARS-CoV-2 Nsp9 with let-7) and **52553** (^1^H,^15^N-HSQC chemical shift assignment of SARS-CoV-2 Nsp9).

## References

[1] Zhu N, Zhang D, Wang W, et al. A novel coronavirus from patients with pneumonia in China, 2019. N Engl J Med. 2020;382(8):727–733. doi: 10.1056/NEJMoa2001017 31978945 PMC7092803

[2] Zhou P, Yang X-L, Wang X-G, et al. A pneumonia outbreak associated with a new coronavirus of probable bat origin. Nature. 2020;579(7798):270–273. doi: 10.1038/s41586-020-2012-7 32015507 PMC7095418

[3] Kim J-M, Chung Y-S, Jo HJ, et al. Identification of coronavirus isolated from a patient in Korea with COVID-19. Osong Public Health Res Perspect. 2020;11(1):3. doi: 10.24171/j.phrp.2020.11.1.02 32149036 PMC7045880

[4] Menachery VD, Graham RL, Baric RS. Jumping species—a mechanism for coronavirus persistence and survival. Curr Opin Virol. 2017;23:1–7. doi: 10.1016/j.coviro.2017.01.002 28214731 PMC5474123

[5] Snijder E, Decroly E, Ziebuhr J. The nonstructural proteins directing coronavirus RNA synthesis and processing. Adv Virus Res. 2016;96:59–126. 27712628 10.1016/bs.aivir.2016.08.008 PMC7112286

[6] Sola I, Almazán F, Zúñiga S, et al. Continuous and discontinuous RNA synthesis in coronaviruses. Annu Rev Virol. 2015;2(1):265–288. doi: 10.1146/annurev-virology-100114-055218 26958916 PMC6025776

[7] Park GJ, Osinski A, Hernandez G, et al. The mechanism of RNA capping by SARS-CoV-2. Nature. 2022;609:793–800. doi: 10.1038/s41586-022-05185-z 35944563 PMC9492545

[8] Kim D, Lee J-Y, Yang J-S, et al. The architecture of SARS-CoV-2 transcriptome. Cell. 2020;181(4):914–921. e910. doi: 10.1016/j.cell.2020.04.011 32330414 PMC7179501

[9] Gautret P, Lagier J-C, Parola P, et al. Hydroxychloroquine and azithromycin as a treatment of COVID-19: results of an open-label non-randomized clinical trial. Int J Antimicrob Agents. 2020;56(1):105949. doi: 10.1016/j.ijantimicag.2020.105949 32205204 PMC7102549

[10] Gao J, Tian Z, Yang X. Breakthrough: chloroquine phosphate has shown apparent efficacy in treatment of COVID-19 associated pneumonia in clinical studies. Biosci Trends. 2020;14(1):72–73. doi: 10.5582/bst.2020.01047 32074550

[11] Chen L, Xiong J, Bao L, et al. Convalescent plasma as a potential therapy for COVID-19. Lancet Infect Dis. 2020;20(4):398–400. doi: 10.1016/S1473-3099(20)30141-9 32113510 PMC7128218

[12] Hossein-Khannazer N, Shokoohian B, Shpichka A, et al. Novel therapeutic approaches for treatment of COVID-19. J Mol Med. 2020;98(6):789–803. doi: 10.1007/s00109-020-01927-6 32494931 PMC7268974

[13] Gómez-Carballa A, Pardo-Seco J, Pischedda S, et al. Sex-biased expression of the TLR7 gene in severe COVID-19 patients: insights from transcriptomics and epigenomics. Environ Res. 2022;215:114288. doi: 10.1016/j.envres.2022.114288 36152884 PMC9508271

[14] Mantovani S, Daga S, Fallerini C, et al. Rare variants in Toll-like receptor 7 results in functional impairment and downregulation of cytokine-mediated signaling in COVID-19 patients. Genes Immun. 2022;23(1):51–56. doi: 10.1038/s41435-021-00157-1 34952932 PMC8703210

[15] Lehmann SM, Krüger C, Park B, et al. An unconventional role for miRNA: let-7 activates Toll-like receptor 7 and causes neurodegeneration. Nat Neurosci. 2012;15(6):827–835. doi: 10.1038/nn.3113 22610069

[16] Zealy RW, Wrenn SP, Davila S, et al. microRNA‐binding proteins: specificity and function. Wiley Interdiscip Rev: RNA. 2017;8(5):e1414. doi: 10.1002/wrna.1414 28130820

[17] Min KW, Zealy RW, Davila S, et al. Profiling of m6A RNA modifications identified an age‐associated regulation of AGO 2 mRNA stability. Aging Cell. 2018;17(3):e12753. doi: 10.1111/acel.12753 29573145 PMC5946072

[18] Min K-W, Jo MH, Shin S, et al. AUF1 facilitates microRNA-mediated gene silencing. Nucleic Acids Res. 2017;45(10):6064–6073. doi: 10.1093/nar/gkx149 28334781 PMC5449627

[19] Littler DR, Gully BS, Colson RN, et al. Crystal structure of the SARS-CoV-2 non-structural protein 9, Nsp9. Iscience. 2020;23(7):101258. doi: 10.1016/j.isci.2020.101258 32592996 PMC7282741

[20] Maeta M, Kataoka M, Nishiya Y, et al. RNA polymerase II subunit D is essential for zebrafish development. Sci Rep. 2020;10(1):13213. doi: 10.1038/s41598-020-70110-1 32764610 PMC7413394

[21] Yoon J-H, Jo MH, White EJ, et al. AUF1 promotes let-7b loading on argonaute 2. Genes Dev. 2015;29(15):1599–1604. doi: 10.1101/gad.263749.115 26253535 PMC4536308

[22] Yoon JH, Abdelmohsen K, Kim J, et al. Scaffold function of long non-coding RNA HOTAIR in protein ubiquitination. Nat Commun. 2013;4(1):2939. doi: 10.1038/ncomms3939 24326307 PMC4556280

[23] Kim HH, Kuwano Y, Srikantan S, et al. HuR recruits let-7/RISC to repress c-Myc expression. Genes Dev. 2009;23:1743–1748. doi: 10.1101/gad.1812509 19574298 PMC2720259

[24] Gordon DE, Jang GM, Bouhaddou M, et al. A SARS-CoV-2 protein interaction map reveals targets for drug repurposing. Nature. 2020;583(7816):459–468. doi: 10.1038/s41586-020-2286-9 32353859 PMC7431030

[25] Jeong JS, Jiang L, Albino E, et al. Rapid identification of monospecific monoclonal antibodies using a human proteome microarray. Mol Cell Proteomics. 2012;11(6):O111.016253. doi: 10.1074/mcp.O111.016253 PMC3433917 22307071

[26] Min KW, Choi KM, Mun H, et al. Mature microRNA-binding protein QKI suppresses extracellular microRNA let-7b release. J Cell Sci. 2024;137(21). doi: 10.1242/jcs.261575 PMC11574364 39308343

[27] Mun H, Lee S, Choi S, et al. Targeting of CYP2E1 by miRnas in alcohol-induced intestine injury. Mol Cells. 2024;47(7):100074. doi: 10.1016/j.mocell.2024.100074 38901530 PMC11267015

[28] Rüdel S, Wang Y, Lenobel R, et al. Phosphorylation of human argonaute proteins affects small RNA binding. Nucleic Acids Res. 2011;39(6):2330–2243. doi: 10.1093/nar/gkq1032 21071408 PMC3064767

[29] Min KW, Davila S, Zealy RW, et al. eIF4E phosphorylation by MST1 reduces translation of a subset of mRNAs, but increases lncRNA translation. Biochim Biophys Acta Gene Regul Mech. 2017;1860(7):761–772. doi: 10.1016/j.bbagrm.2017.05.002 28487214

[30] Miknis ZJ, Donaldson EF, Umland TC, et al. Severe acute respiratory syndrome coronavirus nsp9 dimerization is essential for efficient viral growth. J Virol. 2009;83(7):3007–3018. doi: 10.1128/JVI.01505-08 19153232 PMC2655571

[31] Groban ES, Narayanan A, Jacobson MP, et al. Conformational changes in protein loops and helices induced by post-translationalphosphorylation. PLOS Comput Biol. 2006;2(4):e32. doi: 10.1371/journal.pcbi.0020032 16628247 PMC1440919

[32] Chatterjee S, Ade C, Nurik CE, et al. Phosphorylation induces conformational rigidity at the C-terminal domain of AMPA receptors. J Phys Chem B. 2019;123(1):130–137. doi: 10.1021/acs.jpcb.8b10749 30537817 PMC6465090

[33] Khushman M, Bhardwaj A, Patel GK, et al. Exosomal markers (CD63 and CD9) expression pattern using immunohistochemistry in resected malignant and nonmalignant pancreatic specimens. Pancrease. 2017;46(6):782–788. doi: 10.1097/MPA.0000000000000847 PMC5494969 28609367

[34] Lin Y, Zhang C, Xiang P, et al. Exosomes derived from HeLa cells break down vascular integrity by triggering endoplasmic reticulum stress in endothelial cells. J Extracell Vesicles. 2020;9:1722385. doi: 10.1080/20013078.2020.1722385 32128072 PMC7034510

[35] Guichard A, Lu S, Kanca O, et al. A comprehensive Drosophila resource to identify key functional interactions between SARS-CoV-2 factors and host proteins. Cell Rep. 2023;42(8):112842. doi: 10.1016/j.celrep.2023.112842 37480566 PMC10962759

[36] Minakhina S, Steward R. Melanotic mutants in Drosophila: pathways and phenotypes. Genetics. 2006;174(1):253–263. doi: 10.1534/genetics.106.061978 16816412 PMC1569781

[37] Egloff M-P, Ferron F, Campanacci V, et al. The severe acute respiratory syndrome-coronavirus replicative protein nsp9 is a single-stranded RNA-binding subunit unique in the RNA virus world. Proc Natl Acad Sci. 2004;101(11):3792–3796. doi: 10.1073/pnas.0307877101 15007178 PMC374323

[38] Nikolov D, Burley S. RNA polymerase II transcription initiation: a structural view. Proc Natl Acad Sci. 1997;94(1):15–22. doi: 10.1073/pnas.94.1.15 8990153 PMC33652

[39] Guo J, Price DH. RNA polymerase II transcription elongation control. Chem Rev. 2013;113(11):8583–8603. doi: 10.1021/cr400105n 23919563 PMC4294624

[40] Li S, Lian SL, Moser JJ, et al. Identification of GW182 and its novel isoform TNGW1 as translational repressors in Ago2-mediated silencing. J Cell Sci. 2008;121(24):4134–4144. doi: 10.1242/jcs.036905 19056672 PMC7695043

[41] Biegel JM, Henderson E, Cox EM, et al. Cellular DEAD-box RNA helicase DDX6 modulates interaction of miR-122 with the 5′ untranslated region of hepatitis C virus RNA. Virology. 2017;507:231–241. doi: 10.1016/j.virol.2017.04.014 28456022 PMC5549679

[42] Croce CM. Causes and consequences of microRNA dysregulation in cancer. Nat Rev Genet. 2009;10(10):704–714. doi: 10.1038/nrg2634 19763153 PMC3467096

[43] O’Connell RM, Kahn D, Gibson WS, et al. MicroRNA-155 promotes autoimmune inflammation by enhancing inflammatory T cell development. Immunity. 2010;33(4):607–619. doi: 10.1016/j.immuni.2010.09.009 20888269 PMC2966521

[44] Cullen BR. Viral and cellular messenger RNA targets of viral microRNAs. Nature. 2009;457(7228):421–425. doi: 10.1038/nature07757 19158788 PMC3074184

[45] Kincaid RP, Sullivan CS, Hobman TC. Virus-encoded microRNAs: an overview and a look to the future. PLoS Pathog. 2012;8(12):e1003018. doi: 10.1371/journal.ppat.1003018 23308061 PMC3534370

[46] Zhu Y, Zhang Z, Song J, et al. SARS-CoV-2-encoded MiRNAs inhibit host type I interferon pathway and mediate allelic differential expression of susceptible gene. Front Immunol. 2021;12:767726. doi: 10.3389/fimmu.2021.767726 35003084 PMC8733928

[47] Zhang S, Amahong K, Sun X, et al. The miRNA: a small but powerful RNA for COVID-19. Brief Bioinform. 2021;22(2):1137–1149. doi: 10.1093/bib/bbab062 33675361 PMC7989616

[48] Merino GA, Raad J, Bugnon LA, et al. Novel SARS-CoV-2 encoded small RNAs in the passage to humans. Bioinformatics. 2020;36(24):5571–5581. doi: 10.1093/bioinformatics/btaa1002 PMC7717134 33244583

[49] Thapar R. Structural basis for regulation of RNA-binding proteins by phosphorylation. ACS Chem Biol. 2015;10:652–666. doi: 10.1021/cb500860x 25535763 PMC4372107

[50] Furuichi Y, LaFiandra A, Shatkin AJ. 5′-terminal structure and mRNA stability. Nature. 1977;266(5599):235–239. doi: 10.1038/266235a0 557727

[51] Dunckley T, Parker R. The DCP2 protein is required for mRNA decapping in Saccharomyces cerevisiae and contains a functional MutT motif. Embo J. 1999;18(19):5411–5422. doi: 10.1093/emboj/18.19.5411 10508173 PMC1171610

[52] Stevens A. mRNA-decapping enzyme from Saccharomyces cerevisiae: purification and unique specificity for long RNA chains. Mol Cell Biol. 1988;8(5):2005–2010. doi: 10.1128/MCB.8.5.2005 2838740 PMC363379

[53] Mizrahi O, Nachshon A, Shitrit A, et al. Virus-induced changes in mRNA secondary structure uncover cis-regulatory elements that directly control gene expression. Mol Cell. 2018;72(5):862–874.e865. doi: 10.1016/j.molcel.2018.09.003 30318442

[54] McNab F, Mayer-Barber K, Sher A, et al. Type I interferons in infectious disease. Nat Rev Immunol. 2015;15(2):87–103. doi: 10.1038/nri3787 25614319 PMC7162685

[55] Arunachalam PS, Wimmers F, Mok CKP, et al. Systems biological assessment of immunity to mild versus severe COVID-19 infection in humans. Science. 2020;369(6508):1210–1220. doi: 10.1126/science.abc6261 32788292 PMC7665312

[56] Gomez-Carballa A, Pardo-Seco J, Pischedda S, et al. Sex-biased expression of the TLR7 gene in severe COVID-19 patients: insights from transcriptomics and epigenomics. Environ Res. 2022;215:114288. doi: 10.1016/j.envres.2022.114288 36152884 PMC9508271

[57] Diebold SS, Kaisho T, Hemmi H, et al. Innate antiviral responses by means of TLR7-mediated recognition of single-stranded RNA. Science. 2004;303(5663):1529–1531. doi: 10.1126/science.1093616 14976261

[58] Titos I, Juginović A, Vaccaro A, et al. A gut-secreted peptide suppresses arousability from sleep. Cell. 2023;30(7):1382–1397.e21. doi: 10.1016/j.cell.2023.02.022 PMC10216829 36958331

[59] Yoon JH, Abdelmohsen K, Srikantan S, et al. LincRNA-p21 suppresses target mRNA translation. Mol Cell. 2012;47(4):648–655. doi: 10.1016/j.molcel.2012.06.027 22841487 PMC3509343

[60] Kwon SH, Liu KD, Mostov KE. Intercellular transfer of GPRC5B via exosomes drives HGF-mediated outward growth. Curr Biol. 2014;24:199–204. doi: 10.1016/j.cub.2013.12.010 24412205 PMC3938203

[61] Sonoda H, Lee BR, Park KH, et al. miRNA profiling of urinary exosomes to assess the progression of acute kidney injury. Sci Rep. 2019;9(1):4692. doi: 10.1038/s41598-019-40747-8 30886169 PMC6423131

[62] Trott O, Olson AJ. AutoDock Vina: improving the speed and accuracy of docking with a new scoring function, efficient optimization, and multithreading. J Comput Chem. 2010;31(2):455–461. doi: 10.1002/jcc.21334 19499576 PMC3041641

[63] O’Boyle NM, Banck M, James CA, et al. Open babel: an open chemical toolbox. J Cheminform. 2011;3(1):33. doi: 10.1186/1758-2946-3-33 21982300 PMC3198950

[64] Hu S, Xie Z, Onishi A, et al. Profiling the human protein-DNA interactome reveals ERK2 as a transcriptional repressor of interferon signaling. Cell. 2009;139(3):610–622. doi: 10.1016/j.cell.2009.08.037 19879846 PMC2774939

[65] Huang da W, Sherman BT, Lempicki RA. Bioinformatics enrichment tools: paths toward the comprehensive functional analysis of large gene lists. Nucleic Acids Res. 2009;37(1):1–13. doi: 10.1093/nar/gkn923 19033363 PMC2615629

[66] Huang da W, Sherman BT, Lempicki RA. Systematic and integrative analysis of large gene lists using DAVID bioinformatics resources. Nat Protoc. 2009;4(1):44–57. doi: 10.1038/nprot.2008.211 19131956

[67] Delaglio F, Grzesiek S, Vuister GW, et al. Nmrpipe: a multidimensional spectral processing system based on UNIX pipes. J Biomol NMR. 1995;6(3):277–293. doi: 10.1007/bf00197809 8520220

[68] Lee W, Rahimi M, Lee Y, et al. POKY: a software suite for multidimensional NMR and 3D structure calculation of biomolecules. Bioinformatics. 2021;37(18):3041–3042. doi: 10.1093/bioinformatics/btab180 33715003 PMC8479676

[69] F Dudás E, Puglisi R, Korn SM, et al. Backbone chemical shift spectral assignments of SARS coronavirus-2 non-structural protein nsp9. Biomol NMR Assign. 2021;15(2):235–241. doi: 10.1007/s12104-021-10011-0 33755914 PMC7985572

[70] Lee W, Cornilescu G, Dashti H, et al. Integrative NMR for biomolecular research. J Biomol NMR. 2016;64(4):307–332. doi: 10.1007/s10858-016-0029-x 27023095 PMC4861749

[71] Rahimi M, Chiu A, Estefania Lopez Giraldo A, et al. REDEN: interactive multi-fitting decomposition-based NMR peak picking assistant. J Magn Reson. 2024;358:107600. doi: 10.1016/j.jmr.2023.107600 38039655

[72] Eastman P, Swails J, Chodera JD, et al. OpenMM 7: rapid development of high performance algorithms for molecular dynamics. PLOS Comput Biol. 2017;13(7):e1005659. doi: 10.1371/journal.pcbi.1005659 28746339 PMC5549999

[73] Shen Y, Bax A. Protein backbone and sidechain torsion angles predicted from NMR chemical shifts using artificial neural networks. J Biomol NMR. 2013;56(3):227–241. doi: 10.1007/s10858-013-9741-y 23728592 PMC3701756

