# Supplementary figures and images for: SARS-CoV-2 RNA-binding protein suppresses extracellular miRNA release
Source: RNA Biol. 2025 Jul 1;22(1):1–17. doi: 10.1080/15476286.2025.2527494 (PMC12239786; doi:10.1080/15476286.2025.2527494)

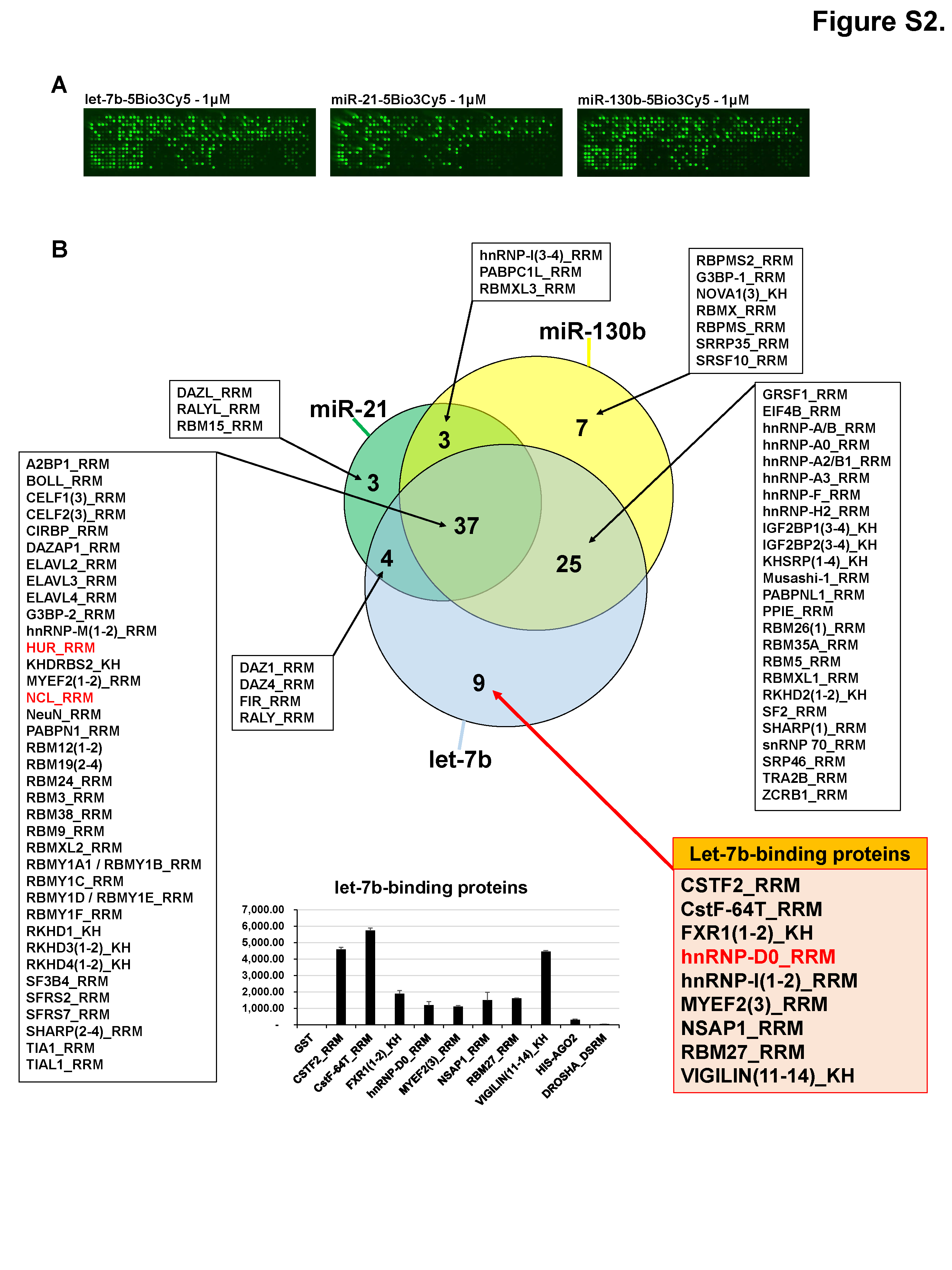

Supplement: NSP9 (Figures) Final_edit_Page_09.tif [file KRNB_A_2527494_SM3515.tif]

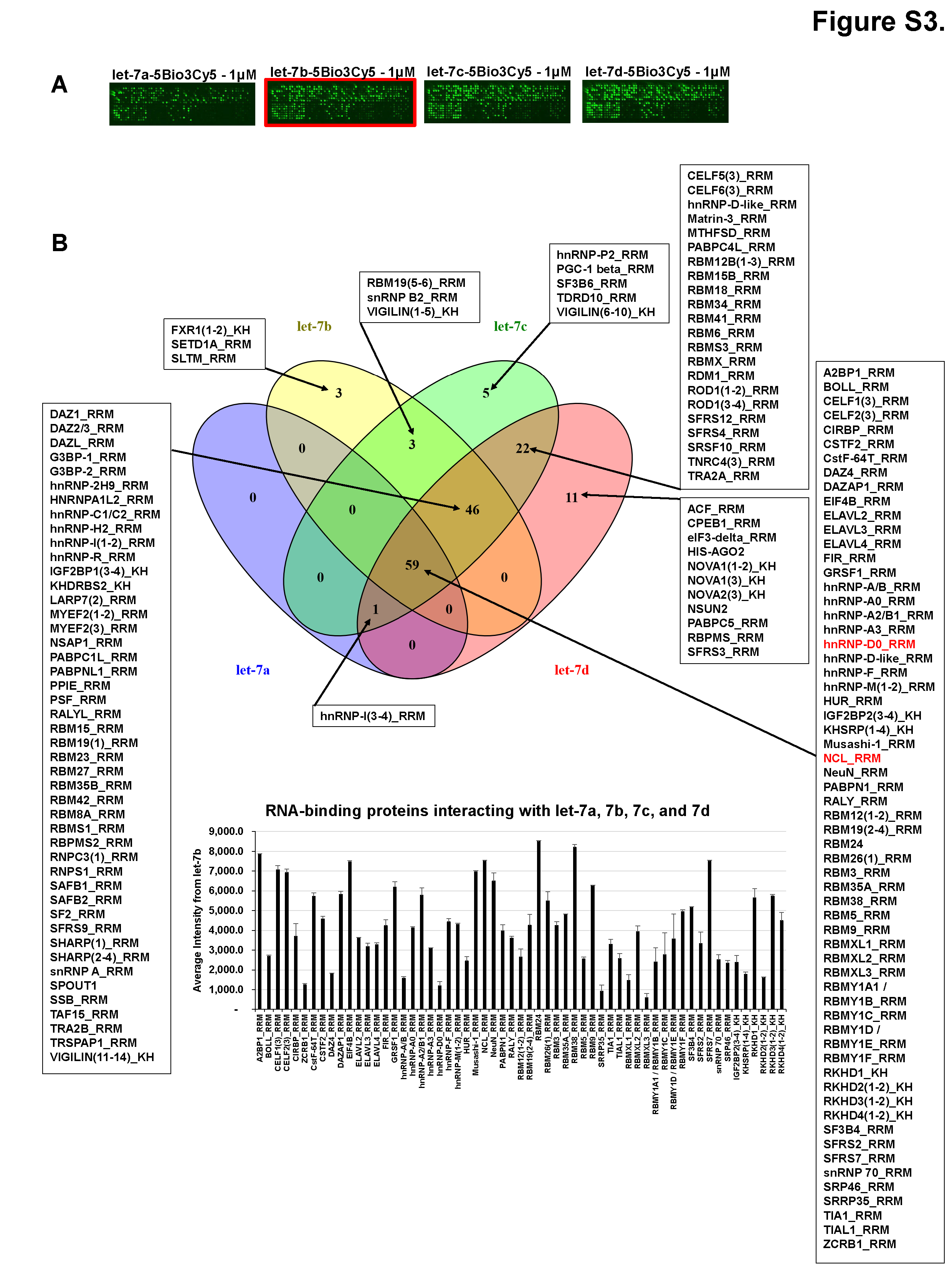

Supplement: NSP9 (Figures) Final_edit_Page_10.tif [file KRNB_A_2527494_SM3514.tif]

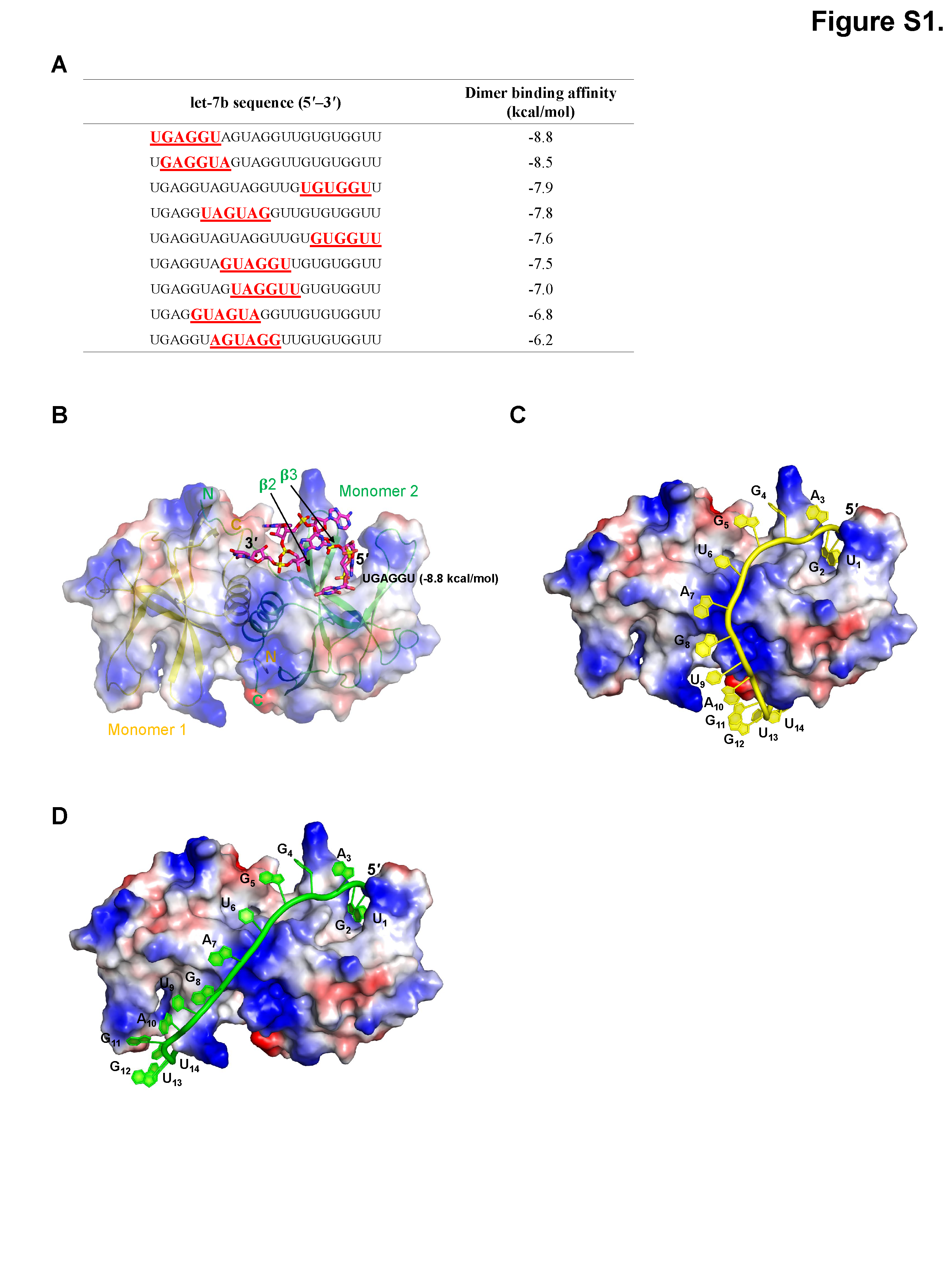

Supplement: NSP9 (Figures) Final_edit_Page_08.tif [file KRNB_A_2527494_SM3512.tif]
